# Calibration of Cohorts of Virtual Patient Heart Models Using Bayesian History Matching

**DOI:** 10.1007/s10439-022-03095-9

**Published:** 2022-10-21

**Authors:** Cristobal Rodero, Stefano Longobardi, Christoph Augustin, Marina Strocchi, Gernot Plank, Pablo Lamata, Steven A. Niederer

**Affiliations:** 1 Cardiac Electro-Mechanics Research Group (CEMRG), Biomedical Engineering and Imaging Sciences Department, King’s College London, London, UK; 2 Cardiac Modelling and Imaging Biomarkers (CMIB), Biomedical Engineering and Imaging Sciences Department, King’s College London, London, UK; 3 Institute of Biophysics, Medical University of Graz, Graz, Austria; 4 BioTechMed-Graz, Graz, Austria

**Keywords:** Gaussian process emulator, Heart model, Statistical shape model, Uncertainty quantification, In-silico trial, Virtual clinical trial

## Abstract

Previous patient-specific model calibration techniques have treated each patient independently, making the methods expensive for large-scale clinical adoption. In this work, we show how we can reuse simulations to accelerate the patient-specific model calibration pipeline. To represent anatomy, we used a Statistical Shape Model and to represent function, we ran electrophysiological simulations. We study the use of 14 biomarkers to calibrate the model, training one Gaussian Process Emulator (GPE) per biomarker. To fit the models, we followed a Bayesian History Matching (BHM) strategy, wherein each iteration a region of the parameter space is ruled out if the emulation with that set of parameter values produces is “implausible”. We found that without running any extra simulations we can find 87.41% of the non-implausible parameter combinations. Moreover, we showed how reducing the uncertainty of the measurements from 10 to 5% can reduce the final parameter space by 6 orders of magnitude. This innovation allows for a model fitting technique, therefore reducing the computational load of future biomedical studies.

**Supplementary Information:**

The online version contains supplementary material available at 10.1007/s10439-022-03095-9.

## Introduction

Model calibration is the process of adjusting the parameters of a model to maximize the agreement between observed data and simulations. Patient-specific cardiac models are being used to prospectively guide therapies^2^ and retrospectively to perform in-silico trials.^24^ Both these applications require the calibration of a large number of models, before proceeding with the analysis of the results of the simulations.

As computer models are increasingly being used in the clinic, there is a need to improve and accelerate the process of model calibration to patient data.^15^ Moreover, although it is not the end-goal, the acceleration process of parametrization is one of the technical barriers to overcome to be able to scale digital twins to the industrial level.^16^

### Current Limitations of the State of the Art

Classical techniques for calibration of patient-specific cardiac models include sweeping over the parameter space,^13^ genetic algorithms^12^ or multivariate regression or Markov chain Monte Carlo (MCMC).^7^ In Ref. 13, Nasopoulou *et al*. reformulated Guccione's material law for cardiac mechanics to fit two parameters. This fitting involved minimising with respect to two cost functions using a sweeping of the space and was therefore highly expensive. Genetic algorithms are also used for parameter fitting. In Ref. 12, Margara *et al*. use this family of algorithms to fit a full electromechanical model, using a multiobjective cost function based on the distance between the estimated parameter and the experimental interval range. One of the main disadvantages of all these approaches is that they do not consider the uncertainty of the estimation. In Ref. 7, Johnstone *et al*. consider the uncertainty of the calibration using an MCMC approach to calibrate electrophysiology models, but with the inherent cost of this approach. In all the aforementioned techniques, there is a need for a high number of evaluations of the models. Each patient case is treated independently, so the cost of calibrating each new patient remains constant. This limits their utility in calibrating cohorts due to their prohibitive cost.

Surrogate models offer a low-cost alternative to a full model evaluation when performing calibration but they require the evaluation of full simulations to generate a training data set. For instance, physics-Informed Neural Networks (PINNS)^3^ have been proposed as surrogates for models, accelerating the model evaluation by up to 30 times. However, PINNS lack a method for tracking the uncertainty of the surrogate model. A different choice for surrogate models is Gaussian Process Emulators (GPEs).^11^ Using GPEs we can rapidly evaluate a function that approximates a costly simulation and obtain the uncertainty inherent to the emulator as well. In Ref. 23 there was no parameter fitting, but rather using literature values for the parameters, all of them fixed, without personalisation. In this work we show how to accelerate the fitting process to use patient-specific data instead of literature-based. In Ref. 17 Noè *et al*. use GPEs to emulate left-ventricular contraction and then fit the Holzapfel–Ogden model by minimising a loss function that takes into account the uncertainty of the emulator. In this approach however a single solution is provided for the combination of parameters while multiple solutions could be viable, and the applicability to a cohort is limited since the authors apply this method to a single mesh.

### Purpose of This Work

In this work we propose two important innovations: 1) representing the patient anatomy as a set of scalar variables, since this allows all simulations for all patients to be defined in a common parameter space, and 2) using simulations used to calibrate previous patients to accelerate the calibration of new patients.

We wish to investigate the feasibility of Bayesian History Matching (BHM) for repeated calibration of a standard modelling framework to new data for a new patient. This paper provides an implementation of this approach and verifies that this conceptual method can work using clinically relevant anatomies and simulations where we know the ground truth parameters. This provides a high throughput method to calibrate patient-specific anatomy and material properties at clinical timescales.

**Figure 1:**
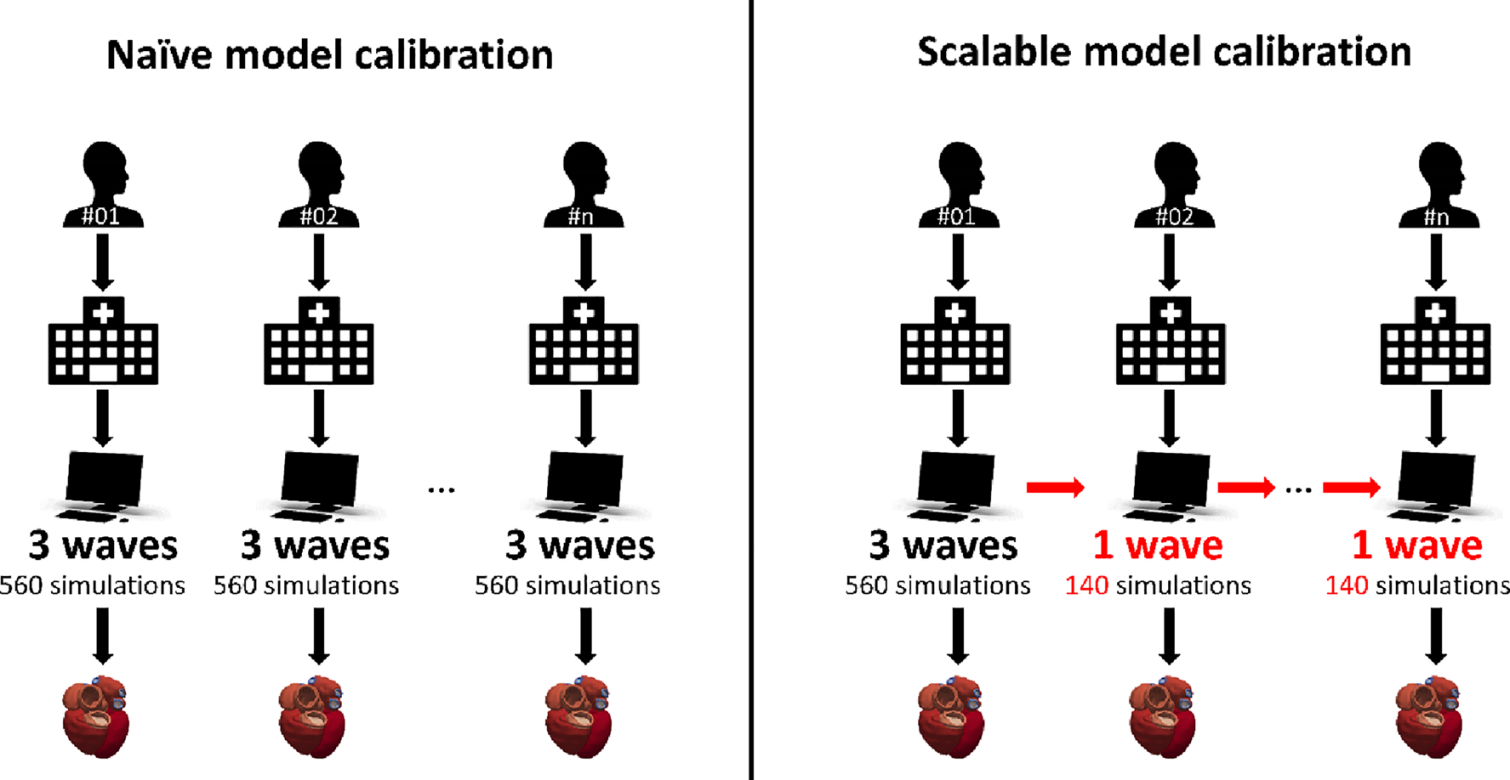
Conceptual abstract of the classical approach to model calibration (”naïve”) vs our approach (“scalable”). The term “wave” refers to an iteration for BHM but it applies to other calibration methods. The main difference is to reuse the previously done calibration on a different patient to reduce the number of simulations run.

A conceptual abstract of the motivation is presented in Fig. 1.

### Paper Structure

In this work, we first give some background details on the elements needed to perform BHM, including the meshes used for the shape model, the shape model itself, and the simulations run. We also provide information about the surrogate models used (GPEs) and the biomarkers analysed. We used the evaluations of the surrogate models to perform two different applications: a calibration of the models using purely literature data and to demonstrate how this can be used to calibrate multiple models serially. In the latter case, we first estimate the cost of calibrating 4 independent models. We then show how the cost of calibrating 2 models is reduced when calibrating them serially. We also test the effect of calibrating two more models with highly dissimilar anatomy. Lastly, we investigate the effect of the uncertainty assumed in the computational models, showing how data acquisition with more accuracy could improve the precision of model calibration.

## Materials and Methods

In many cardiac calibration studies,^11^ a mesh is generated and then the parameters are fitted. Here we propose to represent the anatomy, and hence the mesh, by the weights of the SSM. In this way, the mesh becomes scalar parameters that need to be fit, and the whole model can be represented as a vector of scalars. This allows us to build a single emulator over this vector, that applies to all models for all patients..

In this section, we describe the methods to build meshes from the SSM and run the EP simulations, as well as details about the emulators and the BHM.

### Generation of Models and Simulation Data

Anatomical meshes are described by the modes of a Statistical Shape Model (SSM), described previously in.^23^ Briefly, the SSM used is based on 19 meshes derived from patient CT images. PCA was then applied to the distance from a target mesh and the template to calculate the main directions (modes) of variability. New meshes can be created as a linear combination of the modes added to the template. The SSM had a total of 18, but each mesh was described by the weights of the first 9 modes only, which explain 89.99% of the total variance, setting the rest to 0. The bounds for the initial parameter space of the 9 modes were constrained to be the smallest interval containing the modes of the CT cohort defined in Ref. 23.

EP simulations were performed on the biventricular mesh of each case using the finite element framework Cardiac Arrhythmia Research Package (CARP),^1,26^ built upon extensions of the openCARP EP framework^20^ (http://www.opencarp.org). The reaction-eikonal model^14^ was used, including fibre architecture and a fast endocardial conduction (FEC) layer modelling the Purkinje network. The parameters modified are the fibre angle ($\alpha )$, the FEC layer height, the conduction velocity in the fibre direction ($CV$) and two scalars for the relative conduction velocity with respect to the fibre direction: one in the cross-fibre direction (${k}_{xf}$) and one in the FEC layer (${k}_{FEC}$).

More details on the SSM and the EP simulations including the rationale for the bounds of the parameter values and the ranges for the 9 SSM modes can be found in the Supplementary Material.

Clinical data is inherently noisy and sparse. This means that we may not always have the quantity and quality of data that we need to calibrate a model. We would like to create models that capture this uncertainty and can in turn propagate this uncertainty forward into model predictions. By identifying the plausible parameter ranges that can explain the data, we quantify this uncertainty and can reflect this in model predictions. This will be key for the creation and translation of credible patient specific models.^6^

A summary of the material and structural parameter values is shown in Table 1.

**Table 1:** Functional parameters used in the EP simulations, with the initial range tested.

Parameter	Initial range	Units
Fibre angle ($\alpha$)	[40, 90]	$^\circ$
FEC layer height	[33, 100]	% of the apico-basal extension
$CV$	[0.64, 0.92]	m/s
${k}_{xf}$	[0.11, 0.35]	–
${k}_{FEC}$	[1.1, 8.75]	–

This provides an approximation for the parameter space for all plausible healthy human heart electrophysiology models. We propose to use a combination of GPE and BHM to identify the region within this 14-dimensional space that yields the parameter values for the simulations that are plausible explanations for patient data observations. This approach accounts for both uncertainty due to errors in the GPE and errors in observations.

### Gaussian Process Emulators

To accelerate the evaluation of the different values for the parameters through the simulations, we used GPEs as surrogates for each simulation output of interest. Each GPE ${\varvec{f}}$ (one per output feature) was defined as

$$ {\varvec{f}}\left( {\varvec{x}} \right)\;\underline{\underline{{{\text{def}}}}}\; m\left( {\varvec{x}} \right) + {\mathcal{G}}\left( {\varvec{x}} \right) + {{\varvec{\upvarepsilon}}} $$

where ${\varvec{x}}=({x}_{1},\dots , {x}_{n})$ is the vector of input parameters, $m(\cdot )$, also called a “mean” function is a linear interpolation:

$$m\left({\varvec{x}}\right)={\beta }_{0}+\sum {\beta }_{i}{x}_{i},$$

and $\mathcal{G}\left(\cdot \right)$ is a zero-mean Gaussian Process with a covariance matrix, also called “kernel”, with the form

$$  K\left( {\user2{x},\user2{x^{\prime}}} \right) = \sigma _{f}^{2}  \cdot \exp \left( { - \mathop \sum \limits_{i} \left( {\frac{{x_{i}  - x_{i} ^{\prime } }}{{\delta _{i}^{2} }}} \right)^{2} } \right). $$

This specific kernel is also known as the radial basis function (RBF) kernel. Lastly, the term ${\varvec{\upvarepsilon}}$ corresponds to Gaussian noise with zero mean and variance ${\sigma }_{n}$.

This formulation satisfies desirable properties (in the noise-free case) such as having zero variance in the training points and having less variance when the evaluated point is close to a previously trained point.

The parameters to optimise are then the vectors of weights ${\varvec{\beta}}=({\beta }_{1} ,\dots ,{\beta }_{n})$ and length-scale${\varvec{\delta}}=({\delta }_{1},\dots ,{\delta }_{n})$; and the scalars of bias${\beta }_{0}$, output-scale ${\sigma }_{f}$ and noise variance${\sigma }_{n}$. The GPE training is performed as described previously.^10^ All the hyper-parameters are fitted to maximize the log-marginal likelihood (more details in Ref. 22).

The accuracy of a GPE was evaluated using both the coefficient of determination ${R}^{2}$ and the independent standard error:

$$\mathrm{ISE}=\#\left\{\Vert \overline{{\varvec{f} }\left({\varvec{x}}\right)}-\overline{{\varvec{y}} }\Vert <2 \cdot SD\left({\varvec{f}}\left({\varvec{x}}\right)\right)\right\},$$

where # represents the cardinal (number of elements), ${\varvec{y}}$ is the output of the simulation with ${\varvec{x}}$ as input, the overline indicates the average value and $SD\left(\cdot \right)$ indicates the standard deviation. Intuitively, the ISE metric takes into account the distance between the simulation prediction to the target observed value but also takes into account the uncertainty of the emulator. Therefore, if the real observed value falls within 2 SD of the predicted output, it will be counted as a “success”.

### Bayesian History Matching

To fit the models and reduce the initial range of parameter values, we followed a BHM strategy as reported previously.^10^ BHM is an iterative method, wherein each iteration (or “wave”) a region of the parameter space is ruled out.

The approach has one initialising step that is followed by four steps that are iterated on. To initialise BHM, the first step consists on sampling the initial space, in our case, using Latin hypercube sampling. In the iterative stage, the sampled points are used to run simulations (and generate meshes). Next, a set of GPEs are trained, one for each phenotype, over the full parameter space. The GPE outputs are then compared with observations from data (in our case, patient measurements and literature data) and GPE evaluations that are deemed non-implausible are kept. Lastly, the reduced NROY region is used to sample new points, in our case, using the cloud technique. The process is then repeated.

The criterion to rule it out is if the emulation (with GPEs in our case) with that set of parameter values produces an implausible result. The implausibility score for a vector of parameters ${\varvec{x}}$ is defined as

$$  I^{2} \left( \user2{x} \right) = \mathop {\max }\limits_{{i = 1, \ldots ,m}} \frac{{\left( {\rm{\mathbb{E}}\left[ {\user2{f}_{i} \left( \user2{x} \right)} \right] - \mu _{i} } \right)^{2} }}{{\rm{\mathbb{V}}ar\left[ {\user2{f}_{i} \left( \user2{x} \right)} \right] + \sigma _{i}^{2} }},~ $$

where $m$ is the number of univariate GPEs (one per each output feature), and ${\mu }_{i}\pm {\sigma }_{i}\in {\mathbb{R}}$ represents the observed or desired feature value taken from literature, clinical data or simulations.

This implausibility measure quantifies the discrepancy between the expected and obtained output and also considers the variance of the data. High values of $I({\varvec{x}})$ imply that ${\varvec{x}}$ is unlikely to give a good match (${\varvec{x}}$ is implausible), but low values mean that ${\varvec{x}}$ is a non-implausible candidate parameter set. Due to this, the current parameter region in each wave is defined as a “not-ruled-out-yet” (NROY) region.

**Figure 2:**
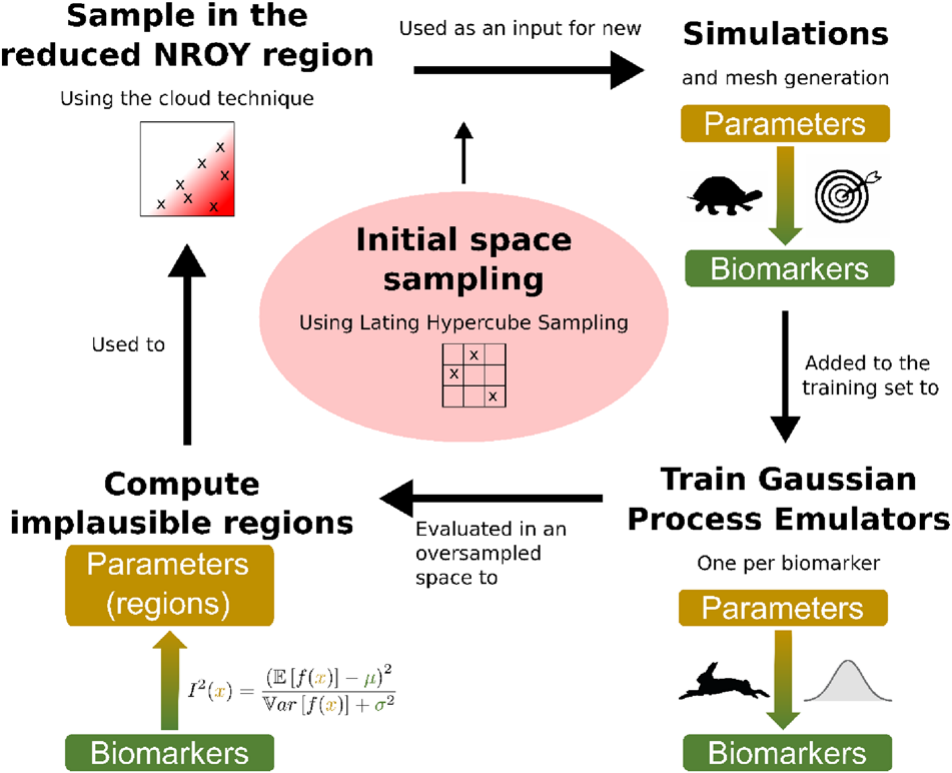
Graphical abstract of one wave of Bayesian History Matching (BHM). “NROY” stands for “not-ruled-out-yet”, ${\varvec{f}}({\varvec{x}})$ refers to the emulation of the point ${\varvec{x}}$ and $\mu \pm \sigma$ corresponds to the clinical variability observed for the biomarker.

A summary of the steps of one wave are described in Fig. 2.

To calibrate using simulation data, the variance term (${\sigma }_{i}$) is not obtained as the simulated results are deterministic (and individual) simulations. Nonetheless, setting to 0 that term might not be an appropriate option since the models and simulations themselves always carry extra uncertainty. Moreover, that choice would raise the value of the implausibility, potentially discarding regions that could be compatible with the results of the simulations if these were modified slightly. To tackle this problem, we set as an initial estimation the SD to be 10% of the corresponding mean value, (${\sigma }_{i}=0.1\cdot {\mu }_{i}$). This value is in agreement with those reported by Ref. 9, where the minimum value of SD over the mean is 0.1. To assess how much the final space would be reduced if we modify the uncertainty of the simulations, we repeated the analysis setting the SD to be 5% of the corresponding mean value, (${\sigma }_{i}=0.05\cdot {\mu }_{i}$).

The initial NROY space for the first wave is sampled using Sobol’ sequences^25^ as the space-filling experimental design. For each following wave, a subset of 140 points from the current NROY space is simulated using the full model. These results are added to previous simulation results and the GPE is then retrained. With each wave the GPE is expected to become more accurate in the NROY space, thus allowing the NROY to be reduced further. Additional NROY points can be generated using the “cloud” technique^5^ if too few points remain for the next wave. Briefly, for every point in NROY space, we generate new points by sampling from a multinormal distribution centred on that point and “expanding” further out with a scaling factor. Intuitively, the idea is to look closer at the borders between the NROY region and the implausible space. A constant number of 10 thousand points were emulated in all the waves.

The final implausibility threshold is aimed to be 3, based on Pukelsheim’s 3-sigma rule^21^ as previously used in other studies.^10^ In this pilot study, we run BHM for three waves. In the first two waves, we set an implausibility threshold of 3.2. Finally, we run a third wave with an implausibility threshold of 3.

To quantify how the uncertainty of the emulators evolves as we add more waves, we measured the quotient between the variance of the emulator over the variance of the ground truth. Since there is an emulator for every biomarker, we took the maximum value for each of the GPEs, so we have a “worst-case scenario” situation. This produces a value for every point of the space, that we will refer to as the variance quotient. Formally, we have that

$$VQ\left({\varvec{x}}\right)\mathop =\limits^{{{\text{def}}}}  \mathop {\max }\limits_{{i= 1, \ldots m}}   \frac{{\mathbb{V}}ar\left[{{\varvec{f}}}_{i}\left({\varvec{x}}\right)\right]}{{\sigma }_{i}^{2}} $$

is the point-wise variance quotient where ${\varvec{x}}$ is a parameter vector, $m$ being the number of GPEs, ${{\varvec{f}}}_{i}$ a univariate GPE and ${\sigma }_{i}^{2}$ the experimental variance. We therefore will report the maximum and median values of $VQ({\varvec{x}})$ for ${\varvec{x}}\in$ NROY.

We report the NROY space size as a percentage of the original space as well. When comparing different emulators for the same case, we report the percentage of agreement in terms of NROY space and of the whole space (both NROY region and implausible space).

**Figure 3:**
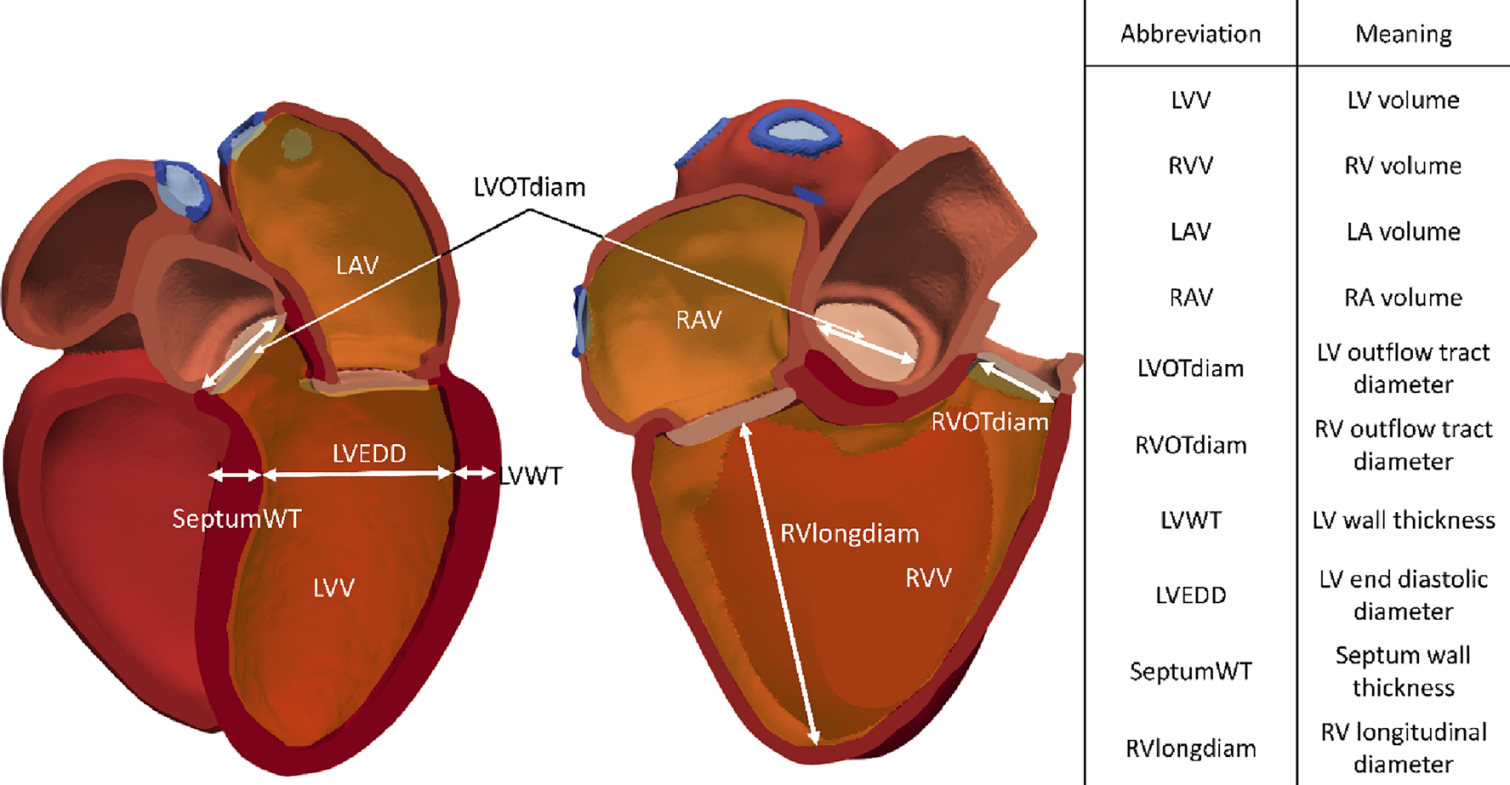
Diagram of the anatomical measurements analysed; LV mass (not shown in the diagram) was also analysed.

In our framework, a GPE was trained for each biomarker or clinical measurement. A diagram of some of the anatomical measurements analysed is shown in Fig. 3.

For the EP simulations, the metrics chosen are the total activation time of both ventricles (TAT) and the total activation time of the left ventricular endocardium (TATLV_endo_). The former would translate almost directly to QRS duration, routinely used in clinical assessments. Although TATLVendo is not measured as often as QRS duration, it has been measured previously in humans^4^ and can help to fit the values of the parameters related to the FEC layer. The target values for TAT and TATLVendo are 76.4 ± 8.2 ms^18^ and 31.3 ± 11.21 ms,^4^ respectively. More details on how the biomarkers are computed from the meshes and the values extracted from literature can be found in the Supplementary Material.

## Results

### Creating a Reference GPE Across Population Parameter Space

To train the GPEs in the initial space (before narrowing it down in successive waves), we ran 438 simulations, with 280, 70 and 88 allocated to training, validation and test sets. The maximum scores were achieved for all the emulators, except for the emulator of the TAT which achieved ${R}^{2}=0.99$ and TATLV_endo_ which achieved an $ISE=98.86$%.

A calibration example using these emulators with literature data can be found in the Supplementary Material.

### Calibration to CT-Based Patient Data

To verify the model calibration process we used simulations to generate synthetic data with known ground truth. We start with the reference GPE calibrated to simulations over the full 14-dimensional parameter space. We first wanted to test the marginal benefit of additional simulations in the NROY space in subsequent waves of BHM.

**Figure 4:**
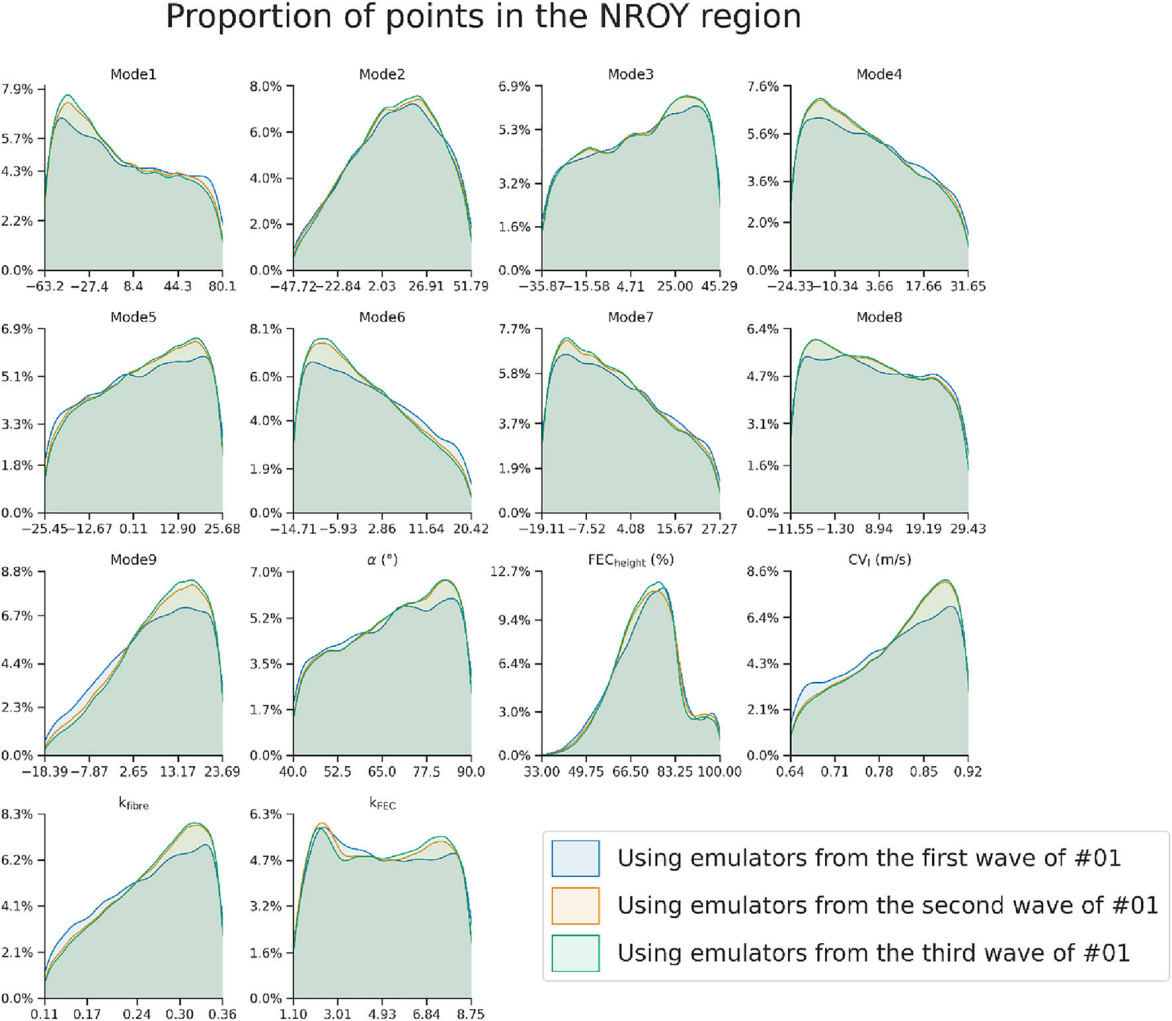
The proportion of points with the values specified in the x-axis in the NROY regions of subject #01 across different waves.

Using the reference population GPE for the first wave, we applied BHM to generate functional and measured anatomical biomarkers from case #01 from the cohort of patients described in.^23^ All the cases used from that cohort are based on CT imaging. We describe the changes in the parameter space with each BHM wave by frequency maps of each parameter as shown in Fig. 4. Applying BHM to the measurements and simulations from case #01 saw the parameter space reduced from 11.24% at wave one to 10.53% after three waves. The accuracy of the emulator decreased from a maximum and median $VQ$ value of 2.59 and 0.93, respectively in the first wave with the reference GPE to 2.92 and 1.11 in the third wave with the patient-specific GPE. The implausibility at the ground truth parameter set is equal to 0.38.

The limited change in the size of the NROY space and distribution of non-implausible parameters indicates that additional simulations in the NROY parameter space in latter waves do not demonstrably improve the GPE. The high accuracy of the reference GPE (${R}^{2}\approx 1$) for all biomarkers means that the GPE does not get meaningfully more accurate with additional simulations in latter waves, hence the parameter space remains nearly unchanged in latter waves.

### Reusing Emulators with a Different Subject

To test the impact of reusing emulators (reusing simulations) for new and unseen patients, we used subjects #01 and #02. Firstly, we ran the BHM pipeline for 3 waves with patient #02. This required an additional 280 simulations, for a total of 560 simulations. In this case, the NROY size was 9.49% of the original space by the first wave, and we achieve a final NROY size of 7.51% of the original space by the third wave. The implausibility for the known target parameter point was 0.2, and the maximum and median $VQ$ were 2.2 and 0.72, respectively.

We then used the emulators from the third wave of subject #01 to fit the model to the biomarkers generated from subject #02. This scenario would mimic the situation of having a bank of simulation data in the clinic and a new patient, for whom there are no previous simulations, needs to be modelled. Using the previously calibrated emulator, we obtained an NROY size of 7.13% of the original size without running any extra waves. This compares with the value of 9.49% in the first wave (no additional simulations) and 7.51% in the final third wave (280 additional simulations). The implausibility of the target ground-truth parameter point was 0.7 which, although higher compared with the full patient-specific pipeline, is still considered non-implausible. The uncertainties were higher, with a maximum and median of 2.99 and 1.38, respectively.

Conversely, we tested the emulators from the third wave of subject #02, but to fit the biomarkers generated from subject #01. Using these already trained emulators, we obtained an NROY size of 8.34% of the original size without running any extra waves. This compares with the value of 11.24% in the first wave (no additional simulations) and 10.53% in the final third wave (280 additional simulations). The implausibility of the target ground-truth parameter point was 0.72. The uncertainties had a maximum and median of 2.51 and 0.82, respectively.

To further understand the similarity of the NROY spaces of the reusing emulators vs running more (patient-specific) waves, we show the match in terms of the NROY region. Using the biomarkers from #01 and the emulators of the third wave of #02 we find a 95.76% match. This means that 95.76% of the points of this NROY (that has a size of 8.34% of the original space) are also in the NROY region of the patient-specific pipeline for subject #01 (that has a size of 10.53% of the original space). Analogously, 97.29% of the points in the NROY when using emulators from #01 and biomarkers from #02 are present in the #02-specific pipeline.

A summary of the statistics for the first two subjects can be found in Table 2.

**Table 2:** Summary of the statistics when using the emulators of #01 and #02 on each other.

	Using biomarkers from subject	Using emulators from #01	Using emulators from #02	Using emulators from the initial wave
NROY size as % of original space	#01	10.53	8.34	11.24
	#02	7.13	7.51	9.49
Implausibility of simulated point	#01	0.38	0.72	0.65
	#02	0.7	0.2	0.47
$VQ$ Max/median	#01	2.92/1.11	2.51/0.82	2.53/0.93
	#02	2.99/1.38	2.2/0.72	2.27/0.82
NROY + RO / NROY match	#01	–	98.7/95.76%	98.09/86.09%
	#02	98.95/97.29%	–	98.02/87.81%

"Using emulators from #” refer to the third wave of each subject. In the scenarios where the emulators and biomarkers come from different patients, no extra waves were run. NROY stands for “Not-ruled-out-yet” region, RO for “ruled-out”. More details on $\mathrm{VQ}$'s meaning in the text. The NROY + RO/NROY match is comparing the specified emulators with the ground truth of running 3 waves on the same subject

A common feature between the two subjects is that when we cross the emulators used and the biomarkers, the NROY size is smaller than if we used the emulators and biomarkers from the same subject. In these cases, having a smaller NROY region does not necessarily mean that the emulators are more accurate. Since the NROY overlap is very high it means that when using emulators and biomarkers of different patients, they do a poor job predicting some of the points that would be in the NROY region using the emulators trained on the data of the same subject as the biomarker.

We repeated the analysis using the emulators trained with the simulations from the initial space, before obtaining a personalised NROY region. This scenario reflects the situation of having an initial emulator trained with data from literature ranges, without specifying anything for any given subject. As can be seen in the last column of Table 2, the results are comparable (only slightly worse) to those trained with more points of the NROY of any specific patient. These results suggest that although running more waves might narrow the NROY space, there is not a qualitative change if we use instead a “global/reference” emulator.

### Reusing Emulators with Very Different Subjects

One of the main caveats that might arise from this pipeline is that reusing emulators might work between similar patients, but if a new subject is too different, the results might not hold. To test this, we ran two more scenarios.

In the first scenario, we computed the $\mathcal{l}1$ distance between the input vector of subject #01 and the input vector of each other subject of the cohort (patients #02-#19). The farthest subject, in this case, was subject #10. Similarly, as the outcome with patient #02, reusing the third wave of patient #01 using the biomarkers of this new subject, lead to similar results as running the full pipeline. The NROY space was 11.5% without running any extra waves in the reusing scenario, compared to 12.2% in the full-pipeline scenario (after three waves); the implausibility of the ground truth point for that subject was 0.39 and $VQ$ had a maximum of 2.44 and a median 1.1 if reusing the simulations for patient #01 compared to an implausibility of 0.34 and $VQ$ with a maximum of 2.02 and median of 1.18 if using the specialised pipeline.

In the second scenario, we computed the $\mathcal{l}1$ distance between the vector of biomarkers of subject #01 and the vector of biomarkers of each other subject in the cohort. The farthest subject, in this case, was subject #18. Using the results of the last wave of #01 with the biomarkers of #18 resulted in a reduction of the original space down to 1.94%. The uncertainty quotient, $VQ$, in the third wave of #18 had a maximum value of 3.64 and a median value of 1.64. The lowest match in terms of the NROY region similarity was also achieved when using the third wave of #01 with the biomarkers of #18: 87.41% of the NROY points in the case of using the emulators of #01 with the biomarkers of #18 had the same status (not implausible) as with the case of running three waves using only case #18.

A summary of all aforementioned statistics for subjects #01, #10 and #18 can be found in Table 3.

**Table 3:** Summary of the statistics when reusing subjects' emulators.

	NROY size as % of original space	Implausibility of simulated point	$VQ$ Max/median	NROY + RO/NROY match
Using emulators from #10 with biomarkers from #01	8.73	0.71	2.43/1.31	99.0/97.67%
Using emulators from #18 with biomarkers from #01	10.82	0.43	3.23/1.32	97.58/97.84%
Using emulators from #01 with biomarkers from #01	10.53	0.38	2.92/1.11	–
Using emulators from #01 with biomarkers from #10	11.5	0.39	2.44/1.1	98.83/97.83%
Using emulators from #10 with biomarkers from #10	12.2	0.34	2.02/1.18	–
Using emulators from #01 with biomarkers from #18	1.94	0.93	3.64/1.64	99.41/87.41%
Using emulators from #18 with biomarkers from #18	2.14	0.48	3.93/1.7	–

When the emulators were used on the biomarkers of the same subject, three waves were run on that subject. Otherwise, the emulators from the third wave of the specified subject were used, without running any extra waves for the new subject. NROY stands for “Not-ruled-out-yet” region, RO for “ruled-out”. More details on the $\mathrm{VQ}$'s meaning in the text

**Figure 5:**
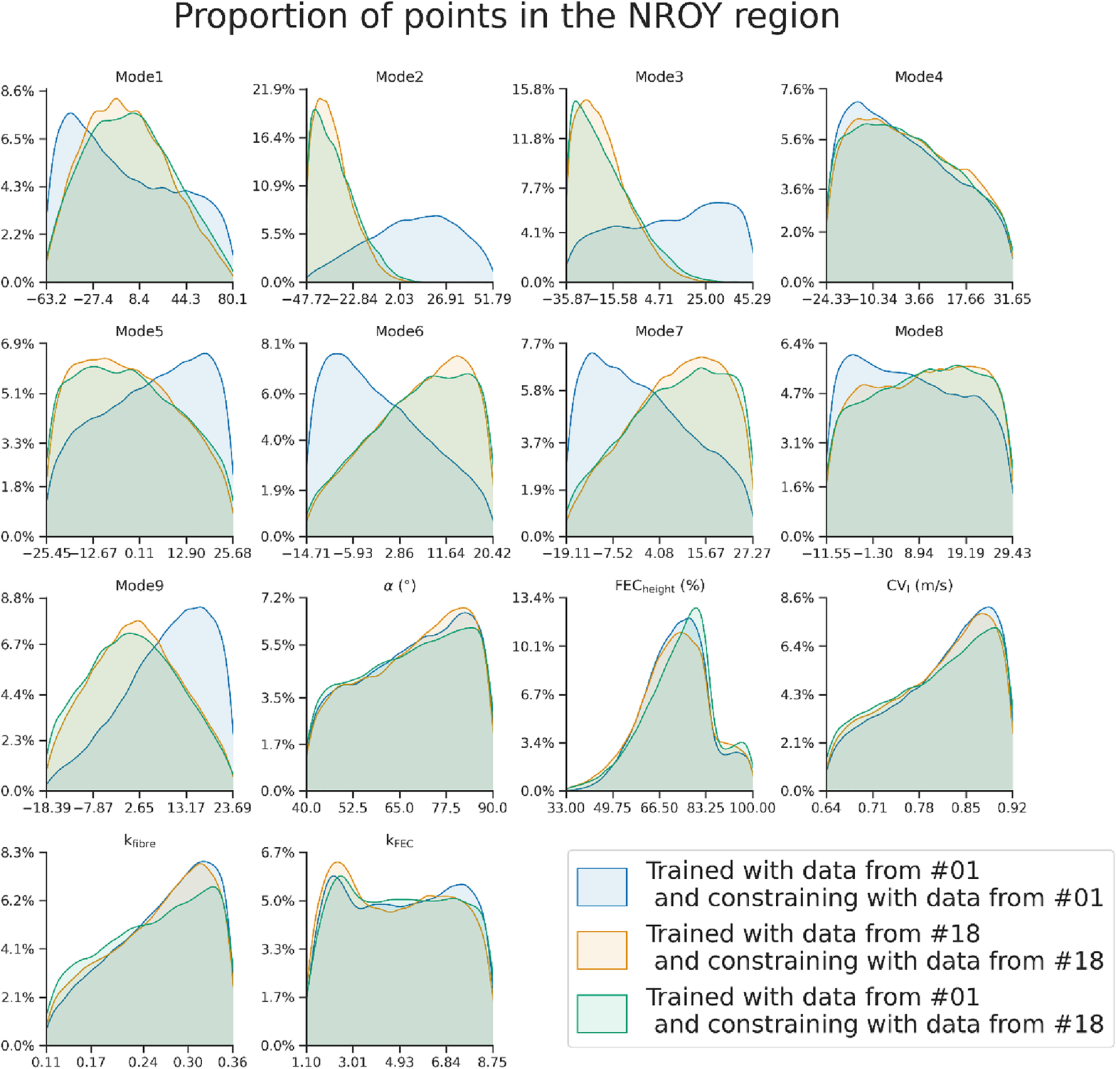
The proportion of points with the values specified in the x-axis in the NROY regions of three different scenarios: using the emulators from the third wave of subject #01 and biomarkers of #01; with the emulators of #18 and the biomarkers of #18 and with the emulators #01 and the biomarkers of #18.

In Fig. 5 we visualize the parameter space of the third wave for subject #01, for subject #18 and using the emulators trained on #01 constrained with the biomarkers from #18. In all the cases, even if the distribution of parameter values is qualitatively different between the two subjects, the distributions when using the biomarkers from #18 follow very similar shapes (in agreement with the NROY match shown in Table 3.

### Reducing the Uncertainty of the Simulations

In all the previous results, we used a prescribed SD term for the data to compute the implausibility of the emulated points. This value (${\sigma }_{i}=0.1\cdot {\mu }_{i}$), was in line with the variance observed in the literature data. We then repeated the analysis but set the SD term to be 5% of the mean value for each biomarker. For the sake of readability, we will refer to cases using ${\sigma }_{i}=0.1\cdot {\mu }_{i}$ as “cases with high uncertainty” and to cases using ${\sigma }_{i}=0.05\cdot {\mu }_{i}$ as “cases with low uncertainty”. A summary of the results can be found in Table 4.

**Table 4:** Comparison of the parameter spaces in the high uncertainty case (H.U.C.) and low uncertainty case (L.U.C.) for the subjects analysed.

Subject	Space overlap (%) between the H.U.C. and the L.U.C	NROY (%) of the L.U.C. present in the NROY of the H.U.C	NROY (%) of the H.U.C. present in the NROY of the L.U.C
#01	90.79	55.14	7.92
#02	92.19	18.36	5.76
#10	88.3	65.4	14.26
#18	97.76	85.82	10.88

In the case of subject #01, the final NROY size went from 10.18% of the original space, in the last wave of the case of high uncertainty, down to 0.43%, in the first wave of the case of low uncertainty. By the third wave, the NROY space is 0.000096% of the original space. Similarly, subject #02’s NROY changed from 7.26 to 0.00003%; subject #10, from 12.2 to 0.0008% and subject #18, from 2.07 to 0.13%.

Since the variance of the subject was decreased, both $VQ$ and the implausibility values will, in general, increase. In terms of $VQ$, the biggest increase was observed in subjects #01 and #02 where the maximum value of $VQ$ changed from 2.2 to 17.34 in the case of subject #02, and the median value changed from 1.11 to 8 in the case of #01.

In terms of the “shape” of the NROY region, we compare the whole parameter space and the NROY regions of the high and low uncertainty cases for the four analysed subjects. We compared the implausibility status of every point (implausible or non-implausible). The highest overlap of the NROY in the low uncertainty case with the NROY of the high uncertainty case was for subject #18, with 85.82% of the non-implausible points in the low uncertainty case being also non-implausible in the high uncertainty case. The lowest percentage of overlap was for subject #02, with 18.36%. We had expected that this value would be closer to 100%.

To have more insight into these effects, we repeated the H.U.C. and the L.U.C. for subject #01 but with an increased initial training set. Instead of having 280 meshes and simulations in the initial space, we added all the simulations ran for subjects #02, #10 and #18 in all the waves. This would be equivalent to using the simulation results from the previous three patients, alongside the reference simulations to calibrate a model for a new patient. Since we ran two more waves for each subject using 140 points in each, the initial space ended with a total of 1120 training points. Using this initial space and running two more waves for subject #01, we get that by the last wave 98.15% of the L.U.C. NROY is included in the H.U.C. NROY, showing that the emulators needed a bigger training set to improve their accuracy.

## Discussion

In this pilot study, we showed how it is possible to reuse results from previous simulations on new, unseen subjects to save computational cost and time on constraining the parameter space and calibrating the models. Although it is not feasible to predict how emulators would behave in any given subject, the tests performed over different subjects (whether from the point of view of the input, or of the output) suggest that as long as they are sufficiently similar (in consideration of both subject characteristics and measurement uncertainty), the calibration will converge to similar parameter spaces.

In classical calibration methods, there is usually a cost function to minimise to fit the parameters of the model. However, this cost function usually involves a comparison with specific biomarkers, such as cardiac work or displacement. In contrast, a fundamental advantage of a GPE + BHM pipeline is that it is robust in the situation where a specific biomarker is not available for a patient or where new measurements are taken.

In terms of computational cost, the cost of a wave of BHM is essentially that of the emulators. As shown in Fig. 1, rather than running multiple simulations every time there is a new case, with this pipeline we build a global/reference emulator that the first time would need a number of simulations to be trained, but as new cases are added, the need of running new simulations is drastically reduced. The cost of evaluating an emulator is virtually null as is essentially a polynomial.

### Performance with a Single Population-Wide Emulator

We showed how the parameter space that describes all electrophysiology and anatomical patient models can be encompassed by a 14-dimension scalar vector. This innovation allows for a global reference GPE to be used in the first wave of a BHM pipeline for any case, without the need of running more simulations. The question is then, would it be enough with a single emulator?

The answer is that this may even be enough if measurements had a large uncertainty. Our experiments using a 10% uncertainty demonstrated only a slight reduction of the NROY size as we run more waves (thus simulations) with the same subject. Fitting the model to population-average parameters we can reduce the NROY space down to 9.97% of the original space so that we know that most cases are going to exist in a parameter subspace. In the case of constraining with patient’s biomarkers, using the emulators trained in the initial space, we obtain NROY sizes of 11.24, 9.49, 14.48 and 2.57% of the original space for subjects #01, #02, #10 and #18, respectively. Running two more extra waves reduced the space to 10.53, 7.51, 12.2 and 2.14%, respectively. This difference is quite small and highlights that the measurement uncertainty of 10% is a quite large one that leads to a similar NROY size for a single patient that is comparable to the whole population level.

Nevertheless, a single population-wide emulator is not enough if measurements have a smaller uncertainty (i.e., a 5% in our experiments). The reason is that the gain of refined emulators in successive waves is potentially very large, with reductions of the NROY size while preserving the ground truth parameter set. The justification to search for an optimal way to refine emulators without the cost of additional simulations is thus clear in the scenario of low uncertainty in the data.

### Limitations

The biomarkers analysed involve only anatomical reconstructions and EP simulations. In more complex scenarios such as mechanics^23^ or computational fluid dynamics simulations,^8^ a reduction of parameters would be needed as a pre-processing step, possibly using the results obtained from the global sensitivity analysis like the ones performed in.^23^

This pilot study is framed in an early stage of the modelling pipeline, and no clinical conclusions can be extracted directly from it. Although the anatomical measurements of the patients used are based on CT imaging, functional data extracted directly from clinical measurements would be needed to create an impact to the clinical community.

The SSM was built over a small cohort (19) of healthy cases. This can affect the results in two ways. First, in a bigger cohort with higher variability, more modes might be needed to explain 90% of the population variability. For instance, in other whole-heart SSMs^19^ with bigger cohorts (*n* = 100) over 30 modes were required to reach 90% of the variance. This, in turn, would increase the input parameter space size and therefore the ability of the emulators to capture the relation between model parameters and biomarkers. And second, the uncertainty of the emulators in our case was in general low, but with more “distant” points (such as diseased cases), this uncertainty would increase, and some extra waves might be needed for personalised cases.

Several parameters remain fixed through this study, and results should be interpreted taking these into account. For instance, the uncertainty of the patient data was set to a fixed percentage of the mean of each biomarker (10 and 5% in the sensitivity analysis). There are parameters qualitatively more influential than others and therefore a change in the uncertainty for this parameter will have a significant effect on the reduction of the NROY space. A biomarker-specific uncertainty might be needed for future approaches.

Lastly, in this pilot study, we set a fixed number of waves with the same thresholds for all the subjects. A natural next step would be to extend this to a convergence-based pipeline where the implausibility threshold is not lowered unless the space reduction (in terms of NROY as % of the initial space) has stabilised.

## Conclusion

In this work, we have presented a pipeline that can accelerate the calibration of cardiac models, an essential step to perform in-silico trials. Moreover, this pipeline can have any input/output combination and can be further applied with any other framework the modelling community.

## Supplementary Information

Below is the link to the electronic supplementary material.

Supplementary file1 (PDF 4312 kb).
