# Supplementary material for: Calibration of Cohorts of Virtual Patient Heart Models Using Bayesian History Matching
Source: Ann Biomed Eng. 2022 Oct 21;51(1):241–52. doi: 10.1007/s10439-022-03095-9 (PMC9832095; doi:10.1007/s10439-022-03095-9)
Supplement: Supplementary file 1 — Supplementary file1 (PDF 4312 kb). [file 10439_2022_3095_MOESM1_ESM.pdf]

Supplementary Material to Calibration of cohorts of virtual patient heart models using Bayesian history matching

Cristobal Rodero[1,2,*], Stefano Longobardi[1], Christoph Augustin[3,4], Marina Strocchi[1], Gernot Plank[3,4], Pablo Lamata[2], Steven A. Niederer[1]

[1] Cardiac Electro-Mechanics Research Group (CEMRG), *Biomedical Engineering and Imaging Sciences Department, King's College London, London, UK*

[2] Cardiac Modelling and Imaging Biomarkers (CMIB), *Biomedical Engineering and Imaging Sciences Department, King's College London, London, UK*

[3] *Institute of Biophysics, Medical University of Graz, Graz, Austria*

[4] *BioTechMed-Graz, Graz, Austria*

*corresponding author. E-mail: cristobal.rodero@kcl.ac.uk

Methods

Generation of new cases

Anatomical meshes are described by the modes of a Statistical Shape Model (SSM), described previously in (Rodero et al., 2021). Briefly, the SSM used is based on 19 meshes derived from CT images. The images were segmented in a semi-automatic manner and tetrahedral meshes were generated including the myocardium of the four chambers, the outflow tracts of the aorta, and the pulmonary vein and rings for the atrial veins to apply suitable boundary conditions for the mechanics' simulations were also added. Using these meshes, an SSM was created relying on an instance of the Large Deformation Diffeomorphic Metric Mapping framework, with the software Deformetrica (Bône, Louis, Martin, & Durrleman, 2018), avoiding a point-to-point registration. In this framework, surfaces are encoded as deRham or mathematical currents. Distances between currents can then be defined with no dependency on specific point coordinates (Vaillant & Glaunès, 2005). Once the distances are computed between the samples, a barycenter shape is extracted. This barycenter or "template", corresponds to the shape that achieves the minimal distance from an optimal transport theory point of view to all initial shapes (Feydy, Charlier, Vialard, & Peyré, 2017). PCA was then applied to the distance from a target mesh and the template to calculate the main directions (modes) of variability. New meshes can be created as a linear combination of the modes added to the template. The SSM had a total of 18, but each mesh was described by the weights of the first 9 modes only, which

explain 89.99% of the total variance, setting the rest to 0. The bounds for the initial parameter space of the 9 modes were constrained to be the smallest interval containing the modes of the CT cohort defined in (Rodero et al., 2021).

Fibres were included in the ventricular myocardium following a rule-based algorithm (J. D. Bayer, Blake, Plank, & Trayanova, 2012). The (longitudinal) fibre direction is defined in the endocardium with the angle α and rotates clockwise throughout the ventricular wall to $-\alpha$ in the epicardium. The angle α is defined as the helical angle with respect to the counterclockwise circumferential direction of the heart when looking from the base towards the apex. The fibres created have a minimum angle of 40° and maximum angle of 90° (Haliot et al., 2019) across all the cases.

To simulate the effect of the Purkinje network, an isotropic fast endocardial conduction (FEC) layer was added to the model as a one-element thick layer (Lee et al., 2019) covering at least the most apical third of the endocardium (Hyde et al., 2015). The extension (or "height") of the FEC layer was also considered as one of the parameters to fit, covering from the most apical third to the whole endocardium (33% to 100% of the endocardium).

EP simulations were performed on the biventricular mesh of each case using the finite element framework Cardiac Arrhythmia Research Package (CARP) (Augustin et al., 2016; Vigmond, Hughes, Plank, & Leon, 2003), built upon extensions of the openCARP EP framework (Plank et al., 2021) (http://www.opencarp.org). The reaction-eikonal model (Neic et al., 2017) was used. Activation was initiated by stimulating the apical third of the endocardium.

The input parameters for the EP model are the conduction velocities (CVs) in the fibre direction, cross-fibre and the FEC layer. To ensure that the CV in the fibre direction was always higher than in the cross-fibre direction and smaller than in the endocardium, instead of using the absolute velocity, an anisotropy ratio with respect to the fibre direction CV was specified instead. The CV in the fibre direction (referred to as CV if not stated otherwise) was bounded to fall between 0.64 (Taggart et al., 2000) and 0.92 m/s (Glukhov et al., 2012); the anisotropy ratio for the cross-fibre direction k_{xf} was bounded to values ranging from 0.11 (Glukhov et al., 2012) to 0.35 and the anisotropy ratio for the FEC layer k_{FEC} was set to range between 1.1 and 8.75 (Durrer et al., 1970; Lee et al., 2019). The upper ranges for the anisotropies were set 20% higher than the values used in (Rodero et al., 2021) to be able to capture the behaviour of emulators around those parameters.

Biomarkers analysed

In our framework, a GPE was trained for each biomarker or clinical measurement. To improve the clinical translation, we chose measurements both obtainable from the meshes/simulations as well as from clinical imaging techniques such as echocardiography. We distinguish between two different scenarios: a first scenario where we use values from previously reported simulations (completely simulated data) to verify that the modelling framework can recover known parameter values when the model perfectly matches the data; and a second scenario

where we used real clinical data from the literature (completely clinical data). In the first scenario, when fitting specific subjects from the CT meshes, we will use the simulations reported previously (Rodero et al., 2021) as the ground truth. In the second scenario, the anatomical biomarkers used are based on the NORRE study (Kou et al., 2014), all of them obtained with echocardiography in healthy adult humans. We only used the measurements affecting the ventricles, except for the chambers' volumes. The analysed anatomical measurements with the corresponding target values are listed in Table 1.

**Biomarkers (abbreviation)**	**Literature values (mean ± SD)**
LV volume (LVV)	92.8 ± 24.8 mL (Kou et al., 2014)
RV volume (RVV)	124 ± 33 mL (Addetia et al., 2018)
LA volume (LAV)	45 ± 13.5 mL (Kou et al., 2014)
RA volume (RAV)	37.5 ± 13.5 mL (Kou et al., 2014)
LV outflow tract diameter (LVOTdiam)	20.3 ± 2.3 mm (Kou et al., 2014)
RV outflow tract diameter (RVOTdiam)	31.9 ± 4.7 mm (Kou et al., 2014)
LV mass	126.8 ± 37.4 g (Kou et al., 2014)
LV end diastolic diameter (LVEDD)	44.3 ± 4.8 mm (Kou et al., 2014)
Septal thickness (SeptumWT)	8.6 ± 1.6 mm (Kou et al., 2014)
RV longitudinal diameter (RVlongdiam)	67.8 ± 8 mm (Kou et al., 2014)
LV end diastolic diameter (LVEDD)	44.3 ± 4.8 mm (Kou et al., 2014)

Table 1. List of anatomical biomarkers analysed. SD stands for standard deviation, LV for "left ventricle", RV for "right ventricle", LA for "left atrium" and RA for "right atrium".

In the meshes generated for each new point in the space, volumes were computed using a 3D version of the Stokes theorem (Zhang, Zhu, & Zhang, 1997), closing the endocardium with the corresponding valves' surfaces and in the case of the atria, with the veins' surfaces, as described previously (Rodero et al., 2021). LVOTdiam and RVOTdiam were calculated from the area of the aortic valve and pulmonary vein surfaces, respectively, assuming a circumferential shape. LV mass was computed as the reference myocardial density of 1.05 g/mL (Vinnakota & Bassingthwaighte, 2004) times the volume of the myocardium calculated as the sum of the volume of the ventricular tetrahedra.

Two regions of interest (ROIs) were defined in the septum and in the LV lateral wall to measure the corresponding thicknesses using universal ventricular coordinates (UVCs) (J. Bayer et al., 2018). The septum ROI was defined as the set of points with an apicobasal coordinate Z between 0.6 and 0.9 (being 0 at the apex and 1 at the base) and with a rotational coordinate

within the central third of the septum. The lateral wall ROI was defined with the same apicobasal values and with the rotational value corresponding to the opposite side of the ventricle ($\pm\pi$). SeptumWT and LVWT were then calculated as the mean distance between the LV endocardium and LV epicardium in the septum or the lateral wall ROI, respectively. LVEDD was calculated as the mean distance between the LV endocardium in the lateral wall ROI and the septal ROI.

RVlongdiam was calculated as the maximum distance from the centroid of the tricuspid valve surface to any point of the RV endocardium.

A diagram of some of the anatomical measurements analysed is shown in Figure 1.

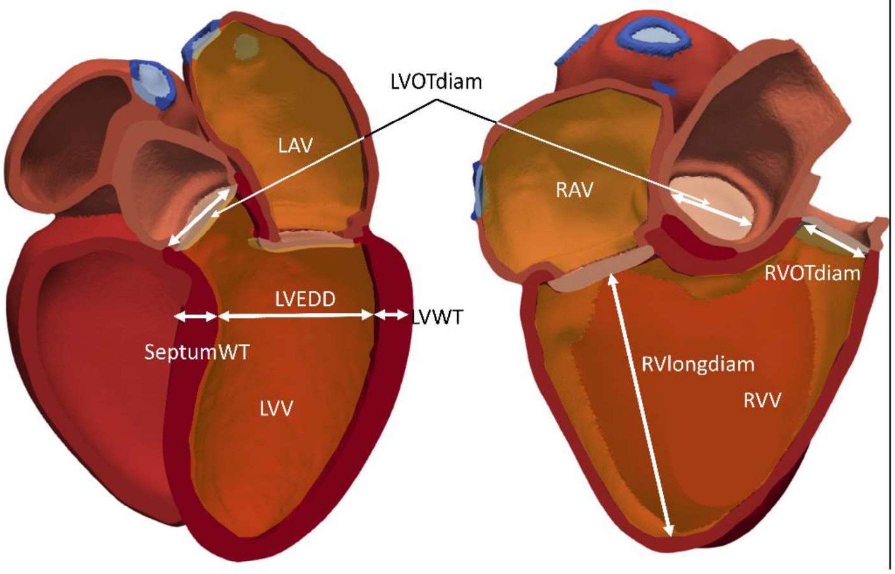

Abbreviation	Meaning
LVV	LV volume
RVV	RV volume
LAV	LA volume
RAV	RA volume
LVOTdiam	LV outflow tract diameter
RVOTdiam	RV outflow tract diameter
LVWT	LV wall thickness
LVEDD	LV end diastolic diameter
SeptumWT	Septum wall thickness
RVlongdiam	RV longitudinal diameter

Figure 1. Diagram of some of the anatomical measurements analysed. LV mass (not shown in the diagram) was also analysed.

For the EP simulations, the metrics chosen are the total activation time of both ventricles (TAT) and the total activation time of the left ventricular endocardium ($TATLV_{endo}$). The former would translate almost directly to QRS duration, routinely used in clinical assessments. Although $TATLV_{endo}$ is not measured as often as QRS duration, it has been measured previously in humans (Cassidy et al., 1984) and can help to fit the values of the parameters related to the FEC layer. The target values for TAT and $TATLV_{endo}$ are 76.4 ± 8.2 ms (Van Oosterom, Hoekema, & Uijen, 2000) and 31.3 ± 11.21 ms (Cassidy et al., 1984), respectively.

Calibration with literature data

As a first experiment, we ran BHM using literature data. We extracted these biomarkers from the NORRE study (Kou et al., 2014), and ran three waves of BHM. By the last wave, the original

space was reduced to 0.88% of the original space size, with a maximum VQ of 3.22, and a median value of 0.6. In Figure 2, we show how the biomarkers obtained when simulating and emulating in the NROY region fall better within the range reported in the literature, as we run more waves.

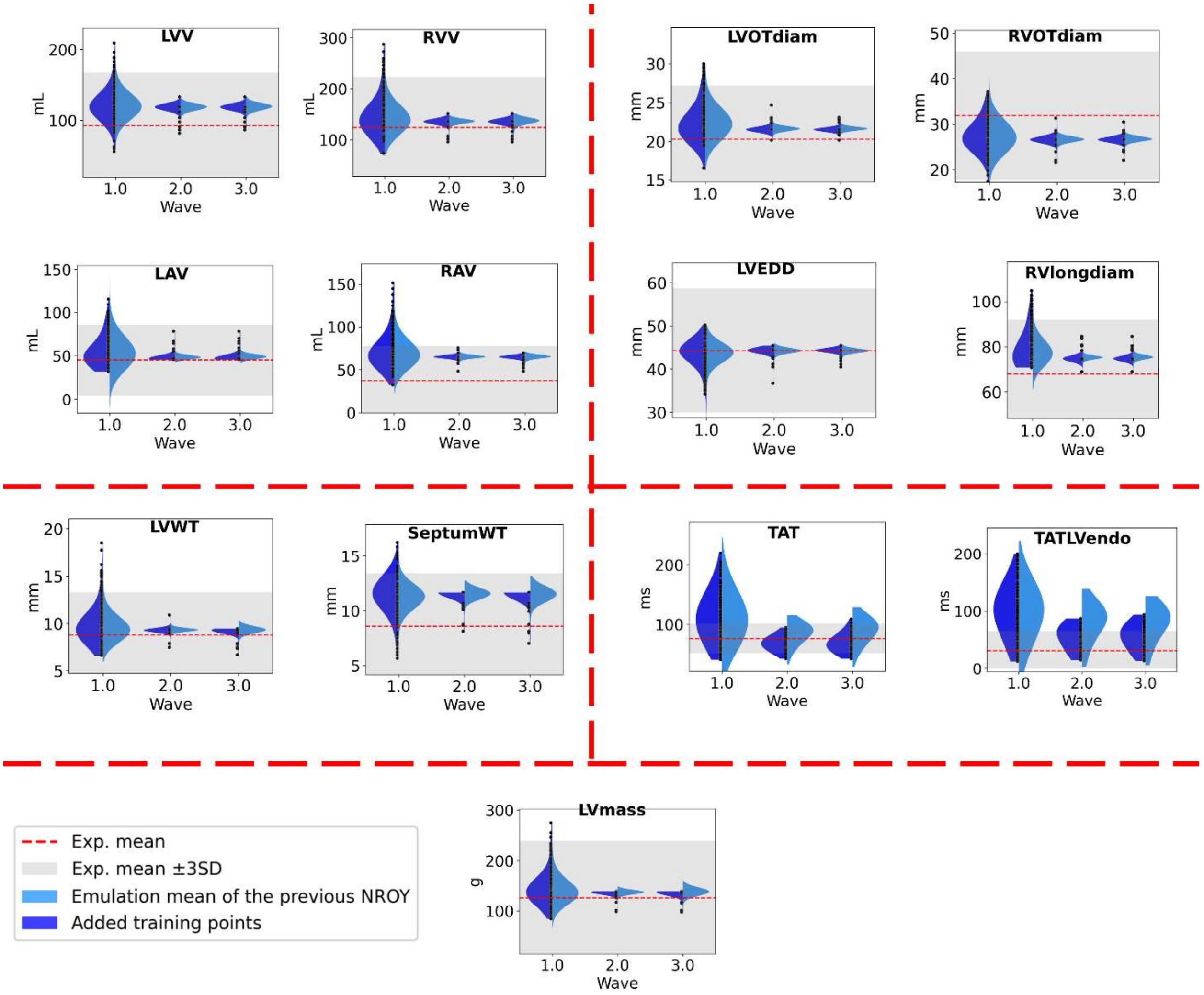

Figure 2. Evolution of the biomarkers for the simulations and emulations in each wave. The black dots are the added simulations for that wave and in dark blue their distribution. "Exp." stands for experimental, meaning the reported literature values, and "NROY" stands for not-ruled-out-yet (region).

Except for the cases of LVEDD and RVOTdiam, in the first wave (when the NROY region is the whole space) part or most of the emulated points have biomarker values outside of the expected range, suggesting that in the initial space there are values not compatible with the expected ranges as reported in the literature. There is not a notable difference between the second and third waves, falling (except in the case of $TATLV_{endo}$) within the target range.

Conflict of interest

The authors declare that they have no conflict of interest.

References

Addetia, K., Maffessanti, F., Muraru, D., Singh, A., Surkova, E., Mor-Avi, V., … Lang, R. M. (2018). Morphologic Analysis of the Normal Right Ventricle Using Three-Dimensional Echocardiography–Derived Curvature Indices. *Journal of the American Society of Echocardiography*, *31*(5), 614–623. https://doi.org/10.1016/J.ECHO.2017.12.009

Augustin, C. M., Neic, A., Liebmann, M., Prassl, A. J., Niederer, S. A., Haase, G., & Plank, G. (2016). Anatomically accurate high resolution modeling of human whole heart electromechanics: A strongly scalable algebraic multigrid solver method for nonlinear deformation. *Journal of Computational Physics*, *305*, 622–646. https://doi.org/10.1016/J.JCP.2015.10.045

Bayer, J. D., Blake, R. C., Plank, G., & Trayanova, N. A. (2012). A Novel Rule-Based Algorithm for Assigning Myocardial Fiber Orientation to Computational Heart Models. *Annals of Biomedical Engineering*, *40*(10), 2243–2254. https://doi.org/10.1007/s10439-012-0593-5

Bayer, J., Prassl, A. J., Pashaei, A., Gomez, J. F., Frontera, A., Neic, A., … Vigmond, E. J. (2018). Universal ventricular coordinates: A generic framework for describing position within the heart and transferring data. *Medical Image Analysis*, *45*, 83–93. https://doi.org/10.1016/j.media.2018.01.005

Bône, A., Louis, M., Martin, B., & Durrleman, S. (2018). Deformetrica 4: An Open-Source Software for Statistical Shape Analysis. *Lecture Notes in Computer Science (Including Subseries Lecture Notes in Artificial Intelligence and Lecture Notes in Bioinformatics)*, *11167 LNCS*, 3–13. https://doi.org/10.1007/978-3-030-04747-4_1

Cassidy, D. M., Vassallo, J. A., Marchlinski, F. E., Buxton, A. E., Untereker, W. J., & Josephson, M. E. (1984). Endocardial mapping in humans in sinus rhythm with normal left ventricles: Activation patterns and characteristics of electrograms. *Circulation*, *70*(1), 37–42. https://doi.org/10.1161/01.CIR.70.1.37

Durrer, D., van Dam, R. T., Freud, G. E., Janse, M. J., Meijler, F. L., & Arzbaecher, R. C. (1970). Total excitation of the isolated human heart. *Circulation*, *41*(6), 899–912. https://doi.org/10.1161/01.CIR.41.6.899

Feydy, J., Charlier, B., Vialard, F.-X., & Peyré, G. (2017). Optimal Transport for Diffeomorphic Registration Optimal Transport for Dif-feomorphic Registration Optimal Transport for Diffeomorphic Registration. *MICCAI*. Retrieved from https://hal.archives-ouvertes.fr/hal-01540455

Glukhov, A. V., Fedorov, V. V., Kalish, P. W., Ravikumar, V. K., Lou, Q., Janks, D., … Efimov, I. R. (2012). Conduction Remodeling in Human End-Stage Nonischemic Left Ventricular Cardiomyopathy. *Circulation*, *125*(15), 1835–1847.

https://doi.org/10.1161/CIRCULATIONAHA.111.047274

Haliot, K., Magat, J., Ozenne, V., Abell, E., Dubes, V., Bear, L., … Bernus, O. (2019). 3D High Resolution Imaging of Human Heart for Visualization of the Cardiac Structure. *Lecture Notes in Computer Science (Including Subseries Lecture Notes in Artificial Intelligence and Lecture Notes in Bioinformatics)*, *11504 LNCS*, 196–207. https://doi.org/10.1007/978-3-030-21949-9_22

Hyde, E. R., Behar, J. M., Claridge, S., Jackson, T., Lee, A. W. C., Remme, E. W., … Niederer, S. A. (2015). Beneficial Effect on Cardiac Resynchronization from Left Ventricular Endocardial Pacing Is Mediated by Early Access to High Conduction Velocity Tissue: Electrophysiological Simulation Study. *Circulation: Arrhythmia and Electrophysiology*, *8*(5), 1164–1172. https://doi.org/10.1161/CIRCEP.115.002677

Kou, S., Caballero, L., Dulgheru, R., Voilliot, D., De Sousa, C., Kacharava, G., … Lancellotti, P. (2014). Echocardiographic reference ranges for normal cardiac chamber size: results from the NORRE study. *European Heart Journal - Cardiovascular Imaging*, *15*(6), 680–690. https://doi.org/10.1093/EHJCI/JET284

Lee, A. W. C., Nguyen, U. C., Razeghi, O., Gould, J., Sidhu, B. S., Sieniewicz, B., … Niederer, S. (2019). A rule-based method for predicting the electrical activation of the heart with cardiac resynchronization therapy from non-invasive clinical data. *Medical Image Analysis*, *57*, 197–213. https://doi.org/10.1016/j.media.2019.06.017

Neic, A., Campos, F. O., Prassl, A. J., Niederer, S. A., Bishop, M. J., Vigmond, E. J., & Plank, G. (2017). Efficient computation of electrograms and ECGs in human whole heart simulations using a reaction-eikonal model. *Journal of Computational Physics*, *346*, 191–211.

Plank, G., Loewe, A., Neic, A., Augustin, C., Huang, Y. L., Gsell, M. A. F., … Vigmond, E. J. (2021). The openCARP simulation environment for cardiac electrophysiology. *Computer Methods and Programs in Biomedicine*, *208*, 106223. https://doi.org/10.1016/J.CMPB.2021.106223

Rodero, C., Strocchi, M., Marciniak, M., Longobardi, S., Whitaker, J., O'Neill, M. D., … Niederer, S. A. (2021). Linking statistical shape models and simulated function in the healthy adult human heart. *PLOS Computational Biology*, *17*(4), e1008851. https://doi.org/10.1371/journal.pcbi.1008851

Taggart, P., Sutton, P. M., Opthof, T., Coronel, R., Trimlett, R., Pugsley, W., & Kallis, P. (2000). Inhomogeneous Transmural Conduction During Early Ischaemia in Patients with Coronary Artery Disease. *Journal of Molecular and Cellular Cardiology*, *32*(4), 621–630. https://doi.org/10.1006/JMCC.2000.1105

Vaillant, M., & Glaunès, J. (2005). Surface Matching via Currents. *Lecture Notes in Computer Science*, *3565*, 381–392. https://doi.org/10.1007/11505730_32

Van Oosterom, A., Hoekema, R., & Uijen, G. J. H. (2000). Geometrical factors affecting the interindividual variability of the ECG and the VCG. *Journal of Electrocardiology*, *33*, 219–227. https://doi.org/10.1054/jelc.2000.20356

Vigmond, E. J., Hughes, M., Plank, G., & Leon, L. J. (2003). Computational tools for modeling electrical activity in cardiac tissue. *Journal of Electrocardiology*, *36 Suppl*, 69–74. Retrieved from http://www.ncbi.nlm.nih.gov/pubmed/14716595

Vinnakota, K. C., & Bassingthwaighte, J. B. (2004). Myocardial density and composition: A basis for calculating intracellular metabolite concentrations. *American Journal of Physiology - Heart and Circulatory Physiology*, *286*(5 55-5). https://doi.org/10.1152/AJPHEART.00478.2003

Zhang, X.-S., Zhu, Y.-S., & Zhang, X.-J. (1997). New approach to studies on ECG dynamics: extraction and analyses of QRS complex irregularity time series. *Med. Biol. Eng. Comput*, *35*, 467–474.
